# Supplementary material for: Distribution and New Records of the Bluntnose Sixgill Shark, Hexanchus griseus (Hexanchiformes: Hexanchidae), from the Tropical Southwestern Atlantic
Source: Animals (Basel). 2022 Dec 26;13(1):91. doi: 10.3390/ani13010091 (PMC9817776; doi:10.3390/ani13010091)
Supplement: Supplementary file 1 [file animals-13-00091-s001.zip › animals-2027432-supplementary.pdf]

Supplementary material

S1. Video of *Hexanchus griseus* caught with handline using line reels near to Saint Peter and Saint Paul's Archipelago

https://www.youtube.com/watch?v=3h3A5QeGD5c

S2. Video of *Hexanchus griseus* landed from artisanal fisheries in Mucuripe Embayment, Ceara state, Brazil.

https://www.youtube.com/watch?v=cxq2t9QfES4
